# Quantitative assessment of ratiometric bimolecular beacons as a tool for imaging single engineered RNA transcripts and measuring gene expression in living cells

**DOI:** 10.1093/nar/gkt561

**Published:** 2013-06-27

**Authors:** Xuemei Zhang, Yang Song, Akash Y. Shah, Virzhiniya Lekova, Arjun Raj, Ling Huang, Mark A. Behlke, Andrew Tsourkas

**Affiliations:** ^1^Department of Bioengineering, University of Pennsylvania, 210 S. 33rd Street, 240 Skirkanich Hall, Philadelphia, PA 19104, USA, ^2^Department of Biology, University of Pennsylvania, 433 S. University Ave, 102 Leidy Laboratories, Philadelphia, PA 19104, USA and ^3^Integrated DNA Technologies, Inc., 1710 Commercial Park, Coralville, IA 52241, USA

## Abstract

Recently, we developed an oligonucleotide-based probe, ratiometric bimolecular beacon (RBMB), which generates a detectable fluorescent signal in living cells that express the target RNA. Here, we show that RBMBs can also be used to image single RNA transcripts in living cells, when the target RNA is engineered to contain as few as four hybridization sites. Moreover, comparison with single-molecule fluorescence *in situ* hybridization confirmed that RBMBs could be used to accurately quantify the number of RNA transcripts within individual cells. Measurements of gene expression could be acquired within 30 min and using a wide range of RBMB concentrations. The ability to acquire accurate measurements of RNA copy number in both HT-1080 cells and CHO cells also suggests that RBMBs can be used to image and quantify single RNA transcripts in a wide range of cell lines. Overall, these findings highlight the robustness and versatility of RBMBs as a tool for imaging RNA in live cells. We envision that the unique capabilities of RBMBs will open up new avenues for RNA research.

## INTRODUCTION

Cell fate, function and phenotype are largely dictated through the control of RNA expression, subcellular localization and processing. This central role of RNA has led to the development and widespread use of various invaluable methods to measure gene expression, including polymerase chain reaction (PCR), microarrays and northern blot. These traditional methods, however, provide only a population average of RNA expression, which can disguise important genetic changes that occur in small subpopulations of cells and ignores cell-to-cell variability. The importance of acquiring information at the single-cell level stems from recent findings that show genetically identical cells under the same environment exhibit diversified phenotypes due to an inherent stochasticity in gene expression (1–4). Emerging technologies such as digital PCR and NanoString® Technologies can provide some insight into RNA expression at the single cells level, but they remain limited in their ability to capture dynamic events and spatial distribution.

Single-molecule fluorescence *in situ* hybridization (smFISH) has also recently emerged as a promising tool that is capable of quantifying RNA copy number in single cells (5). In this method, tens of fluorophore-labeled oligonucleotide probes are hybridized to individual RNA transcripts in fixed cells (6,7). As a result, each transcript appears as a discrete high-intensity fluorescent spot under a fluorescent microscope. smFISH has already provided unique insight into transcriptional bursting (8), phenotypic variability (9) and mRNA stability (10); however, because it requires sample fixation, smFISH is limited in its ability to study RNA dynamics. A complete spatial–temporal profile of RNA expression is expected to be an important advance because it would provide unique insight into mechanisms such as transcriptional bursting (8,11–15), RNA trafficking (16,17) and the dynamic responses of RNA expression to cell stimuli (18). To achieve these capabilities, there has been growing interest in the development of optical probes for imaging of RNA expression in live cells.

**Figure 1.**
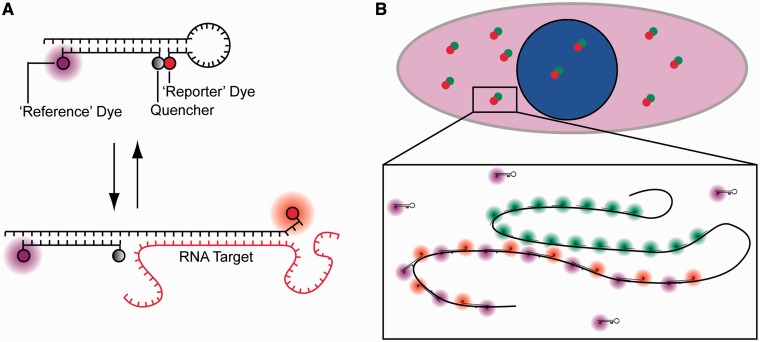
Schematic of RBMBs and the methodology used to assess RBMB performance in cells. (**A**) RBMBs are hairpin-forming oligonucleotide probes that are labeled with a reporter dye, quencher and reference dye. The reporter dye is held in close proximity to the reference dye in the absence of target, leading to a low fluorescent state. Hybridization of the loop-domain to complementary RNA causes the fluorescent dye and quencher to separate, resulting in the restoration of fluorescence. The reference dye remains unquenched regardless of the conformation of the RBMB. The double-stranded domain with a 3′-UU overhang drives nuclear export. (**B**) To evaluate the ability of RBMBs to quantify the number of RNA transcripts in single cells, cells are fixed following the delivery of RBMBs and single-molecule FISH is performed. FISH probes are represented in the schematic as short linear oligonucleotides with a green fluorescent dye, while RBMBs are drawn with a red reporter dye and magenta reference dye. The large number of RBMBs and FISH probes that bind to each RNA transcript results in bright punctate spots within cells, under fluorescence microscopy. Colocalization of RBMB-signal with the smFISH-signal indicates that the RNA transcript was successfully detected by RBMBs.

Perhaps, the most widely adopted probe for live cell imaging is the molecular beacon (MB). MBs are oligonucleotide-based probes that are labeled at one end with a fluorescent reporter and at the other end with a quencher (19,20). In the absence of complementary nucleic acid targets, the MB forms a hairpin structure, which serves to hold the fluorescent reporter and quencher in close proximity. In this configuration, the fluorescence is quenched. In the presence of complementary nucleic acids, hybridization between the central loop of the MB and the target leads to unfolding of the stem and separation of the fluorescent reporter and quencher. In this configuration, fluorescence is restored. Although a number of studies have shown that MBs can be used to detect mRNA in single living cells (21–24), there is growing evidence that the sensitivity of RNA detection is significantly hampered by the sequestration of MBs into the nucleus, where they emit false-positive signals (25–29), and by the large variations in cellular fluorescence that result from heterogeneous intracellular delivery (30). Recently, we developed a new probe for imaging RNA in living cells, ratiometric bimolecular beacons (RBMBs) (Figure 1A), to overcome the limitations of conventional MBs (31). Similar to MBs, RBMBs elicit an increase in reporter fluorescence on hybridization to complementary RNA. However, RBMBs were also designed to possess an 18-base pair double-stranded domain with a 3′-UU overhang and an unquenched reference dye. The unique structure of the RBMB facilitates nuclear export, which dramatically reduces the level of false-positive signals that are detected for at least 24 h, compared with conventional MBs. The reference dye allows for measurements of reporter fluorescence to be adjusted for cell-to-cell variability in RBMB delivery, which allows for more precise measurements of RNA hybridization.

One challenge with designing probes for imaging RNA in live cells is the difficulty in thoroughly assessing their performance. This is largely attributed to the low signal-to-noise that stems from the low copy number of most RNA transcripts, the natural stochasticity of gene expression and the limited brightness of single organic fluorophores. Further, there is a lack of any recognized standard for comparison. In this study, we sought to establish an approach to more quantitatively evaluate the ability of RBMBs to detect RNA transcripts. Specifically, RBMBs were delivered into cells that were engineered to express green fluorescent protein (GFP) RNA transcripts with 96-tandem repeats encoded in the 3′-untranslated region (UTR) (32). On hybridization of the RBMBs to the repeated sequences, single RNA transcripts could be visualized as single bright spots and imaged in real time. At various times following RBMB delivery, the cells were fixed and smFISH was performed against the GFP coding sequence (Figure 1B). This allowed for the colocalization of RBMB and smFISH signals to be quantified. Colocalization was quantified over various RBMB concentrations and at various times after RBMB delivery. In addition, the correlation between RNA copy number and total cellular fluorescence, at lower magnification, was assessed.

## MATERIALS AND METHODS

### Synthesis and design of RBMBs and analogous MBs

RBMBs are composed of two 2′-O-methyl RNA oligonucleotides that are hybridized together. One of the oligonucleotides is labeled with a CF640R (Biotium) reporter dye at the 5′-end and has the sequence 5′-mCmUmUmC mGmUmC mCmAmC mAmAmA mCmAmC mAmAmC mUmCmC mU mGmAmAmG mGmAmC mGmGmC mAmGmC mGmUmG mCmAmG mCmUmC mUmU-3′. Self-complementary domains, which drive the formation of the hairpin structure, are underlined. The second oligonucleotide is labeled at the 5′-end with an Alexa Fluor 750® reference dye and at the 3′-end with an Iowa Black RQ-Sp quencher (Integrated DNA Technologies [IDT]). The sequence of the second oligonucleotide is 5′-mGmAmG mCmUmG mCmAmC mGmCmU mGmCmC mGmUmC-3′. Equal molar ratios of the two oligonucleotides were hybridized in phosphate buffer (48 mM K_2_HPO_4_, 4.5 mM KH_2_PO_4_, 14 mM NaH_2_PO_4,_ pH 7.2) at room temperature overnight. A Superdex 75 prep grade column (GE healthcare, Piscataway, NJ) was used to separate the hybridized RBMBs from single-stranded oligonucleotides and the purified RBMBs were concentrated using a Microcon YM-10 centrifugal device (10 000 MW cutoff, Millipore, Billerica, MA). The final concentration of the RBMBs was determined using a Cary 100 spectrophotometer (Varian, Palo Alto, CA).

A 2′-O-methyl RNA MB, analogous to the RBMB, was also synthesized, 5′-CF640R- mCmUmUmC mGmUmC mCmAmC mAmAmA mCmAmC mAmAmC mUmCmC mU mGmAmAmG-IAbRQSp-3′ (32). A RNA target, complementary to the loop domain of both the RBMB and MB, was synthesized with the sequence 5′-rCrUrC rGrArC rArGrG rArGrU rUrGrU rGrUrU rUrGrU rGrGrA rCrGrA rArGrA rG-3′. All oligonucleotides were synthesized by IDT.

### Thermal and kinetic studies

The thermal denaturation profiles of RBMBs and MBs were obtained by recording fluorescence intensity as a function of temperature using an ABI PRISM 7500 Sequence Detection System (Applied Biosystems, Foster City, CA, USA). RBMB and MB samples consisted of 20 µl hybridization buffer (48 mM K_2_HPO_4_, 4.5 mM KH_2_PO_4_, 14 mM NaH_2_PO_4,_ pH 7.2) with 50 nM of RBMBs/MBs in the absence or presence of 500 nM of complementary RNA target. The temperature was brought to 95°C for 10 min, and reduced by 1°C increment to 25°C. The temperature was held at each temperature increment for 3 min and fluorescence was recorded over the final 30 s. The fluorescence intensity of each test solution was adjusted to correct for the intrinsic variance of fluorescence over temperature. The data were analyzed as previously described (33), using a custom MATLAB program.

The hybridization kinetics between RBMBs/MBs and complementary RNA targets were evaluated using a fluoroMax-3 spectrofluorometer (Jobin Yvon Horiba) equipped with a LFI-3751 AUTOTUNE PID temperature controller (Wavelength Electronics, Bozeman, MT). Specifically, the fluorescence intensity emitted from a 1 ml solution containing 50 nM RBMB was recorded at 37°C as a baseline. To this, 10 µl of 50 µM complementary RNA target was added and rapidly mixed, giving a final concentration of 500 nM target. The fluorescence intensity was recorded at 37°C over time. The data were analyzed as previously described (33), using a custom MATLAB program.

### Preparation of pLenti-dsGFP and pLenti-dsGFP-96mer

The plasmid pTRE-dsGFP-96-mer was kindly provided by Dr Sanjay Tyagi (UMDNJ, New Jersey) (30). This plasmid contains the coding sequence for GFP followed by 96 tandem repeats in the 3′-UTR. The tandem repeats have the following sequence: 5′-CAG GAG TTG TGT TTG TGG ACG AAG AGC ACC AGC CAG CTG ATC GAC CTC GA-3′ (32). RBMBs and MBs were designed to complement a unique sequence within each repeat of the transcribed RNA. Transcription is driven by a tet-off-responsive promoter. The dsGFP-96-mer coding sequence was cloned into the lentiviral expression vector pLenti6/V5 (Invitrogen, Grand Island, NY) by Custom DNA Constructs LLC (University Heights, OH) using the pLenti6/V5 Directional TOPO Cloning Kit (Invitrogen, Grand Island, NY). An analogous construct was made without the 96-mer, i.e. dsGFP. The new plasmids, pLenti-dsGFP-96mer or pLenti-dsGFP, were amplified in stbl2 cells (Invitrogen, Grand Island, NY) and purified using the Qiagen Maxiprep system (Qiagen, Valencia, CA). The sequences of the final plasmid were confirmed by DNA sequencing.

Lentiviral particles containing pLenti-dsGFP-96mer or pLenti-dsGFP were produced using the Virapower Lentiviral Directional TOPO Expression Kit (Invitrogen, Grand Island, NY) according to the manufacturer’s instructions. Briefly, 293FT cells (ATCC, Manassas, VA) were transfected with viral packaging plasmids together with the pLenti-dsGFP-96mer or pLenti-dsGFP vector using Lipofectamine 2000 (Invitrogen, Grand Island, NY). Cell culture media containing virus particles were harvested 72 h after transfection, concentrated using PEG-it Virus Precipitation Solution (System Biosciences, Mountain View, CA) and titered in HT1080 cells.

### Cell culture and cell line construction

The Chinese Hamster Ovary cell line CHO AA8 Tet-Off® was purchase from Clontech (Mountain View, CA). Engineered CHO AA8 Tet-Off® cells expressing pTRE-dsGFP-96-mer (CHO-GFP-96mer) were kindly provided by Sanjay Tyagi (UMDNJ, New Jersey) (32). Cells were cultured in Dulbeco’s MEM media supplemented with 1% Pen/Strep, 10% fetal bovine serum (FBS) and incubated in 37°C incubator with 5% CO_2_.

Human fibrosarcoma cells, HT1080, were purchased from ATCC (ATCC, Manassas, VA), and were cultured in MEM media supplemented with 1% Pen/Strep, 10% FBS and incubated at 37°C incubator with 5% CO_2_. HT1080-GFP-96mer cells were generated by infecting HT1080 cells with pLenti-dsGFP-96mer. Similarly, HT 1080-GFP control cells were generated by infecting HT1080 with pLenti-dsGFP. Infected cells were selected for stable genomic integration using 15 µg/ml blasticidin (Invitrogen, Grand Island, NY). The blasticidin-selected cells were further sorted by flow cytometry in the Flow Cytometry and Cell Sorting facility, University of Pennsylvania.

### Cellular delivery of RBMBs and MBs

The introduction of RBMBs or MBs into cells was achieved by microporation with a Neon transfection system (Invitrogen, Grand Island, NY) as per manufacturer’s protocol. Briefly, a cell pellet containing 300 000 cells was resuspended in 11 µl Resuspension Buffer, supplied with the kit, and mixed with 1 µl of RBMBs (27). The final concentrations of RBMBs are specified in the results section. CHO cells were microporated with 1 pulse for 20 ms at 1700 V and HT1080 cells were microporated with 2 pulses for 25 ms at 950 V. After microporation, cells were washed three times in culture medium and seeded on poly-D-lysine (Sigma, St. Louis, MO) coated eight-well chambered coverglass system (Thermo Scientific, Waltham, MA) for the indicated time period.

### Real-Time PCR

Total RNA from CHO and HT1080 cells was isolated using the High Pure RNA Isolation Kit (Roche, Mannheim, Germany). Total RNA (200 ng) was subsequently reverse-transcribed using a High-Capacity cDNA Reverse Transcription Kit (Applied Biosystems, Foster City, CA). One- to 100-fold diluted cDNA, customized FAM-labeled TaqMan probe sets for GFP or 18 S rRNA (as a control) and the TaqMan universal PCR Master Mix (Applied Biosystems, Foster City, CA) were mixed and the total volume was brought up to 20 µl with deionized water. Real-time PCR (RT-PCR) was performed on an ABI PRISM 7300 Sequence Detection System (Applied Biosystems, Foster City, CA).

### Flow cytometric analysis

Cells were dissociated from culture plates, collected in 1.5 ml centrifuge tubes and washed three times with phosphate buffered saline (PBS) at 1000 RCF for 3 min. Cell pellets were resuspended with PBS at 300 cells/µl and analyzed using a Guava Easycyte Plus system (Guava Technologies, Hayward, CA). Flow cytometry data were analyzed using *FlowJo* software (TreeStar Inc., San Francisco, CA).

### Fluorescent *in situ* hybridization study

Single molecule fluorescent *in situ* hybridization (smFISH) was performed as previously described (7), with minor modifications. The tetramethylrhodamine (TMR)-dsGFP probe, which recognizes the GFP coding sequences and/or Alexa 594–96× probes recognizing the 96 repeated MB targets in 3′-UTR of GFP, were used in smFISH experiments. Cells cultured in eight-well chamber coverglass (50–70% confluency) were washed three times in PBS, fixed with 4% paraformaldehyde for 10 min and permeabilized with 70% ethanol overnight at 4°C. The next day, after equilibrating with wash buffer [5% Formamide, 2× Saline-Sodium Citrate (SSC)] for 2 min, samples were incubated in 50 µl hybridization buffer (5% Formamide, 10% Dextran sulfate, 2× SSC) containing 0.5 µl probes for 3 h at 37°C. The samples were washed with wash buffer for 30 min and incubated with 1:1000 diluted DAPI for another 30 min at 37°C. Cells were washed with 2× SSC and the samples were ready for imaging.

### Image acquisition

All microscopic images were acquired using an Olympus IX81 motorized inverted fluorescence microscope equipped with a back-illuminated EMCCD camera (Andor, UK), a SOLA Light Engine (Lumencor, Beaverton, OR) and a CoolLED pE-100 (740 nm, CoolLED, UK). Images were acquired using the filter sets ET620/60×, ET700/75 m, T660lpxr for CF640R, D350/50, D460/50, 400dclp for DAPI, HQ480/40, HQ535/50, Q505lp for GFP, HQ560/55, HQ645/75, Q595LP for Alexa594, HQ710/75, HQ810/90, Q750LP for Alexa 750 and HQ545/30, HQ620/60, Q570lp for TMR (Chroma, Bellows Falls, VT). A LUC PLAN FLN 40× objective (NA 0.9) or a plan Apo 100× (NA 1.45) oil were used for all imaging studies. An additional 1.6× magnifier was used in combination with the 100× objective, when imaging CHO cells. Metamorph software (Molecular Devices, Sunnyvale, CA) was used to acquire both 2D and 3D images. Three-dimensional image stack were acquired with 0.2 µM increments in the *z*-direction and a total of 56 sections.

### Live Cell Imaging

CHO-GFP-96mer or HT1080-GFP-96mer cells were microporated in the presence of RBMBs (0.4 µM for CHO cells and 0.8 µM for HT1080 cells), plated on poly-D-lysine–coated eight-well chambered coverglass and cultured within a live cell stage top incubation system (Pathology Devices, Westminster, MD). Between 1–5 h after microporation, the Stream Acquisition function in Metamorph software was used to acquire 75 frames for CHO cells and 150 frames for HT1080 cells in the CF640 channel. CHO-GFP-96mer cells were imaged using the 100× objective, while the HT1080-GFP-96mer cells were imaged using the 60× objective. After acquisition, sequential images were opened in Imaris (Bitplane LLC, South Windsor, CT) and saved as an .avi file.

### RNA identification and quantification

Single RNA transcripts were identified in 3D image stacks using a spot detector 3D Laplacian of Gaussian plug-in available for NIH ImageJ (version 1.45 b) (34). All images were subject to a rolling-ball background subtraction before spot detection. To quantify the number of spots, a region of interest (ROI) was hand-drawn around the Z-Projection (Z-Project, Projection Type: Max Intensity) of each cell and applied to the filtered stack, and all local maxima (Find Maxima, Exclude Edge Maxima and Noise tolerance = 0) were identified in each slice of the z-stack. Three-dimensional local maxima were determined from the list of 2D local maxima using MATLAB (Version 7.8.0.347 2009a 64-bit, MathWorks). The list of 2D local maxima was first checked to see whether each maximum’s fluorescence intensity met a user-specified minimum threshold. To determine whether an individual slice 2D local maximum was a 3D local maximum, the intensity of each local maximum in each slice was compared with that of the adjacent pixels in the slices immediately above and below. The region of comparison was a 3 × 3 × 3 cube centered at each local maximum (nine pixels in the above slice, eight surrounding pixels in the same slice, and nine pixels in the slice below). Each 3D local maximum was counted as a single RNA transcript.

### RNA colocalization

The 3D coordinates of RNA transcripts in smFISH and RBMB image stacks were independently identified, according to the above protocol ‘RNA Identification and Quantification’. smFISH and RBMB colocalization was determined using a custom MATLAB program that compares the 3D locations of RNA molecules, as detected by each fluorescent probe. If a FISH-identified RNA molecule was within a user-specified 3D region centered around a RBMB-identified RNA molecule, the two were considered to be colocalized with each other. The 3D colocalization region used for all experiments was a cylinder: four stacks above and below in height, and a circular radius of five pixels. Probe colocalization percentages were calculated for both RBMB and FISH; RBMB colocalization was the number of colocalized probes divided by the total number of RBMB-identified RNA transcripts, and FISH colocalization was the number of colocalized probes divided by the total number of FISH-identified RNA transcripts.

### *In vitro* transcription

pGEM-32mer, pGEM-16mer and pGEM-8mer plasmids, which encode RNA with 32, 16 and 8 RBMB binding sites, respectively, were kindly provided by Dr Sanjay Tyagi (UMDNJ, New Jersey) (32). The pIDTblue-4mer and pIDTblue-2mer plasmids, which encode RNA with 4 and 2 RBMB binding sites, respectively, were purchased from IDT (IDT, Coralville, Iowa). pGEM plasmids were linearized with the restriction enzyme Xmn I (NEB, Ipswich, MA) and pIDTblue plasmids were linearized with Nae I (NEB, Ipswich, MA). Proteins and RNAase were removed by incubating with 0.5% SDS (Life Technologies Corporation, Grand Island, NY) and 0.5 μl proteinase K (Life Technologies Corporation, Grand Island, NY) at 50°C for 30 min. The linear DNA was purified using a PCR purification kit (Qiagen, Valencia, CA). The various RNA sequences were generated by *in vitro* transcription using MAXIscript kit (Life Technologies Corporation, Grand Island, NY) according to the manufacturer’s instructions. Free nucleotides were removed using Illustra MicroSpin G-25 column (GE healthcare, Piscataway, NJ). Fifty microliters of transcribed RNA, together with RBMB (1 µl, 12.5 µM), were hybridized at 37°C overnight. The hybridized RBMB was concentrated to ∼1µl and used for microporation into HT1080 cells. One to 3 h after microporation, live cell streaming movies were acquired using MetaMorph software, as described above. Following the acquisition of movies, cells were fixed by 4% paraformaldehyde and z-stacked images were acquired.

### Quantification of total cellular fluorescence

HT1080-GFP-96mer and CHO-GFP-96mer cells were microporated in the presence of 0.8 µm RBMB, plated into eight-well coverglass chambers, placed in the incubator for 5 h and then fixed. Images of single focal planes in the RBMB reporter channel (CF640R), RBMB-reference dye channel (Alexa750) and TMR channel were then acquired using a 40× objective. Within the same field, z-stacks of individual cells were acquired using a 100× objective, as described above. Images acquired using the 40× objective were stacked and a rolling ball background subtraction was performed. A ROI was hand-drawn around each cell and the total cellular fluorescence (i.e. integrated density) was measured for each image in the stack. For images acquired using the 100× objective, the total number of RNA transcripts detected in the RBMB-reporter channel were quantified as described above.

To approximate cell autofluorescence in RBMB-reporter channel, when using the 40× objective, analogous studies were performed with cells in the absence of RBMBs (i.e. RBMB-negative). Following measurements of total cellular fluorescence in RBMB-negative cells, the correlation between integrated density in CF640R channel and the TMR channel was determined. This relationship was used to estimate the cellular autofluorescence in RBMB-reporter channel in RBMB-positive cells, based on measurements of autofluorescence in the TMR-channel.

The signal enhancement of RBMBs that resulted from hybridzation in individual cells was determined as previously described (31). Briefly, the contribution of RBMB background fluorescence to the total signal in cells was calculated as the product of RBMB-reference fluorescence (RBMB_REF,CELL_) and the fluorescent ratio of RBMB-reporter fluorescence: RBMB-reference fluorescence in the absence of any target RNA (R_CLOSED_). R_CLOSED_ was determined by preparing water-in-oil emulsions, with RBMBs (1 µm) in the water phase, and acquiring fluorescent images of the bubbles using the similar conditions used to image cells (35). The RBMB enhancement signal was then calculated as the difference between the total integrated RBMB-reporter signal (RBMB_REP,CELL_) and the RBMB background signal in cells, as follows:

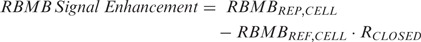



## RESULTS AND DISCUSSION

### Imaging single RNA transcripts with RBMBs in living cells

Following the delivery of RBMBs into HT1080-GFP-96mer cells via microporation, bright fluorescent spots could be readily visualized in living cells by fluorescence microscopy (Supplementary Movie S1). These spots were presumably the consequence of up to 96 RBMBs hybridizing to the 96 tandem repeats inserted into the 3′-UTR of GFP transcripts. This would be consistent with the premise of smFISH, whereby the high local concentration of fluorescent dyes on each individual RNA transcript results in a single bright fluorescent spot. Bright fluorescent spots were not observed in HT1080-GFP and CHO-GFP cells, which do not contain 96 repeats downstream of the GFP coding sequence. The RNA transcripts could be observed moving within the nucleus and cytoplasm. Similar results were obtained following the delivery of RBMBs into CHO-GFP-96mer cells (Supplementary Movie S2).

Imaging the movement of RNA transcripts was generally limited to short time frames (<1 min) because individual fluorescent spots would often move in and out of the focal plane. Rapid 3D imaging could potentially overcome this shortcoming; but the acquisition of each z-stack would likely need to be limited to <0.5 s to ensure the same RNAs could be identified in sequential image stacks. Brighter fluorescent signals could also extend the timeline of live cell imaging studies because RNA transcripts could be detected at further z-distances from the focal plane. This could potentially be achieved by optimizing the spacing between the RBMB hybridization sites. Some quenching of the RBMB reporter dye was evident even following RNA hybridization, likely due to dye–dye and dye–quencher interactions between multiple RBMBs bound to the same RNA.

### Quantitative assessment of RBMB hybridization

**Figure 2.**
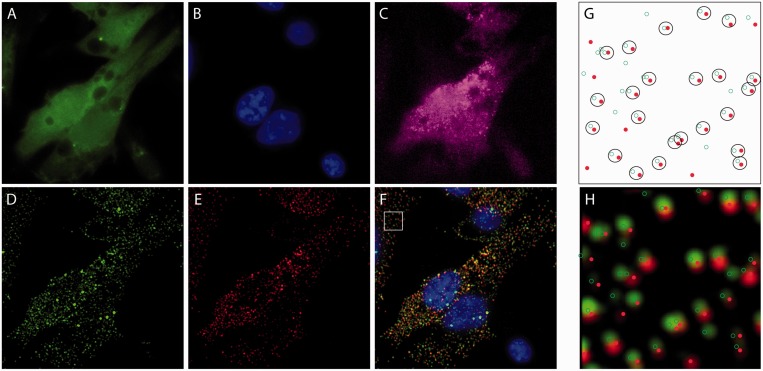
Fluorescent images and analysis of HT1080 cells following RBMB delivery and smFISH. HT1080-GFP-96mer cells were microporated in the presence of 0.8 μM RBMBs and placed in the incubator for 5 h. The cells were then fixed and smFISH was performed. Wide-field fluorescent images of (**A**) GFP, (**B**) DAPI, (**C**) RBMB-reference dye (Alexa 750), (**D**) smFISH probes (TMR) and (**E**) RBMB-reporter dye (CF640R) were acquired. The images of the smFISH probes and the RBMB reporter dye shown are maximum intensity projections of 56-images within a z-stack. (**F**) A merged image that includes DAPI (blue) and the maximum intensity projections of smFISH probes (green) and the RBMB-reporter dye (red). A custom Matlab program was used to identify individual spots in the smFISH and RBMB-reporter channels and to calculate the percent colocalization between the two signals. (**G**) Example Matlab output, showing the smFISH (green circles) and RBMB-reporter (red dots) signals that were detected within the region outlined by a white box in panel F. Spots that were considered to be colocalized are enclosed within a black circle. Notably, the Matlab analysis was performed on the z-stacks (i.e. in three-dimensions) not on the maximum intensity projection images; however, a 2D representation is shown. (**H**) Overlay of the Matlab output and the merged image shows good correspondence between the two.

**Figure 3.**
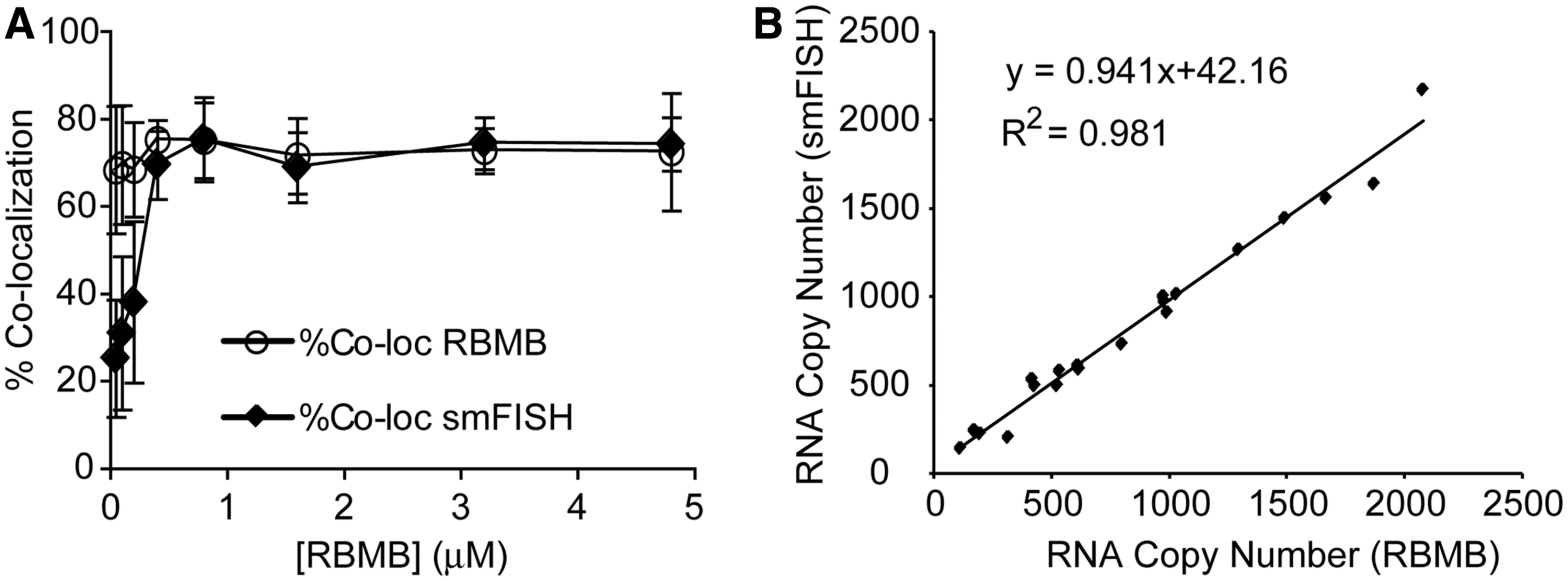
Assessing the accuracy of RBMB-based measurements of RNA copy number. HT1080-GFP-96mer cells were microporated in the presence of RBMBs and placed in the incubator for 5 h. The concentration of RBMBs was varied from 0.05 μM to 4.8 μM. The cells were then fixed and smFISH was performed. (**A**) A custom Matlab program was used to determine the percent of RBMB-reporter signals that were colocalized with smFISH signals and the number of smFISH signals that were colocalized with RBMB-reporter signals, on a cell-by-cell basis. Each data point represents the mean measurement of colocalization (±standard deviation) for at least 20 cells, collected from at least two independent experiments. (**B**) Measurements of RNA copy number per cell, as determined by smFISH and RBMBs, were also compared.

Although RBMBs appeared to be capable of imaging individual RNA transcripts in live cells, it was unclear whether RBMBs could be used to quantify the total number of RNA transcripts, i.e. measure gene expression, in single cells. To address this question, HT1080-GFP-96mer cells were microporated with various concentrations of RBMBs and fixed 5 h after microporation. smFISH was then performed against the coding sequence of GFP RNA. Single wide-field images of DAPI, GFP and fluorescence from the RBMB reference dye were acquired (Figure 2). In addition, z-stacks of the smFISH and RBMB reporter signals were also acquired. Notably, all dyes are spectrally distinct and confirmed to have little to no bleed-through on our system. An overlay of the maximum intensity projections of smFISH and RBMB reporter images revealed a high degree of colocalization for all RBMB concentrations evaluated between 0.4 µm and 4.8 µm. Representative images are provided in Figure 2. Quantitative analysis in three dimensions, using a custom Matlab algorithm, revealed that ∼71–75% of the RBMB reporter signals colocalized with smFISH signals over this range of RBMB concentrations (Figure 3A). Similarly, ∼70–75% of the smFISH signals colocalized with RBMB reporter signals. Moreover, when the total number of RNA transcripts in each individual cell was counted in the smFISH images and RBMB reporter images, there was nearly a perfect correlation (Figure 3B and Supplementary Figure S1). At RBMB concentrations <0.4 µm, the percent of smFISH signals that colocalized with RBMB reporter signals declined with RBMB concentration. This stemmed from a decrease in the intensity of the RBMB reporter signals, which were often below the fluorescence threshold that was implemented.

Control studies where RBMBs were introduced into HT1080-GFP cells (i.e. no 96-repeats) revealed low background fluorescence in the RBMB channel, with only a few spots per cell that surpassed the lower fluorescence threshold, owing to random noise (Supplementary Figure S2). As a positive control, smFISH was performed against both the GFP coding sequence and 96-repeats in the UTR (i.e. two-probe smFISH) in HT1080-GFP-96mer cells. It was found that the colocalization in this case was ∼70–76%, suggesting that RBMBs delivered into live cells perform just as well as smFISH in detecting single RNA transcripts.

The extent of colocalization between smFISH and RBMB reporter fluorescence was also examined in CHO-GFP-96mer cells. The percent co-colocalization was slightly lower in CHO cells and more variable, compared with HT-1080 cells (∼55–71%, Supplementary Figure S3); however, appreciable colocalization was observed with RBMB concentrations as low as 0.1 µm. This is a significantly lower RBMB concentration than could be used with HT-1080 cells. It is hypothesized that this may be due to differences in microporation efficiency between the two cell lines, although differences in cell shape, thickness and cytosolic volume could also play a role.

The more variable degree of colocalization observed in CHO cells appeared to be due to slightly lower image quality than with HT-1080 cells, stemming from the smaller cytoplasmic cross-section and thicker cell body. Thicker cells are generally found to have a higher background signal from unbound RBMBs, due to more out of focal plane fluorescence. This can also explain why lower concentrations of RBMB led to better results—there is less background fluorescence from unbound RBMBs. Nonetheless, these findings suggest that the overall ability of RBMBs to quantify gene expression is not highly sensitive to the experimental conditions and can likely be extended to numerous cell lines.

### Effect of RBMB hybridization on gene expression

**Figure 4.**
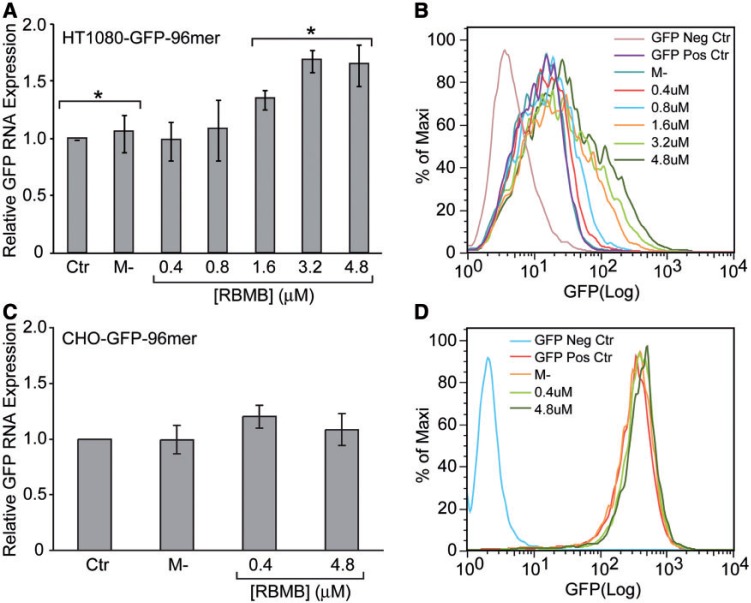
Effect of RBMBs on RNA and protein expression. HT1080-GFP-96mer cells were microporated in the presence of RBMBs and placed in the incubator for 24 h. The concentration of RBMBs was varied from 0.4 to 4.8 μM. (**A**) The level of GFP RNA expression was then evaluated by RT-PCR. Control studies were performed on cells that were microporated in the absence of RBMBs (Ctr) or incubated with RBMBs, but not microporated (M-). (**B**) The level of GFP protein expression was evaluated by flow cytometry. Analagous studies were performed with CHO-GFP-96mer cells. (**C**) The effect of RBMBs on GFP RNA expression in CHO-GFP-96mer cells. (**D**) The effect of RBMBs on GFP protein expression in CHO-GFP-96mer cells. All studies were performed in triplicate. Samples were compared using a two-tailed unpaired *t*-test. An asterisk indicates statistically significant values of *P* < 0.05.

The ability of RBMBs to measure gene expression not only requires accurate detection of RNA transcripts, but the ability to do so without altering RNA stability. Therefore, to assess the affect of RBMBs on gene expression, HT1080-GFP-96mer cells were microporated with various concentrations of RBMBs (0–4.8 µM) and the level of GFP RNA and protein expression were evaluated by RT-PCR and flow cytometry, respectively. It was found that at low RBMB concentrations (≤0.8 µM), there was no noticeable change in either RNA or protein expression (Figure 4A and B, respectively). However, at RBMB concentrations ≥1.6 µM, there was a concentration-dependent increase in both GFP RNA and protein levels. These findings suggest that high RBMB concentrations can lead to RNA stabilization. Interestingly, in CHO-GFP-96mer cells, no increase in GFP RNA or protein levels was observed for any of the RBMB concentrations evaluated (Figure 4C and D, respectively). Therefore, the ability of RBMBs to stabilize RNAs may be cell line dependent.

### Intracellular hybridization kinetics

**Figure 5.**
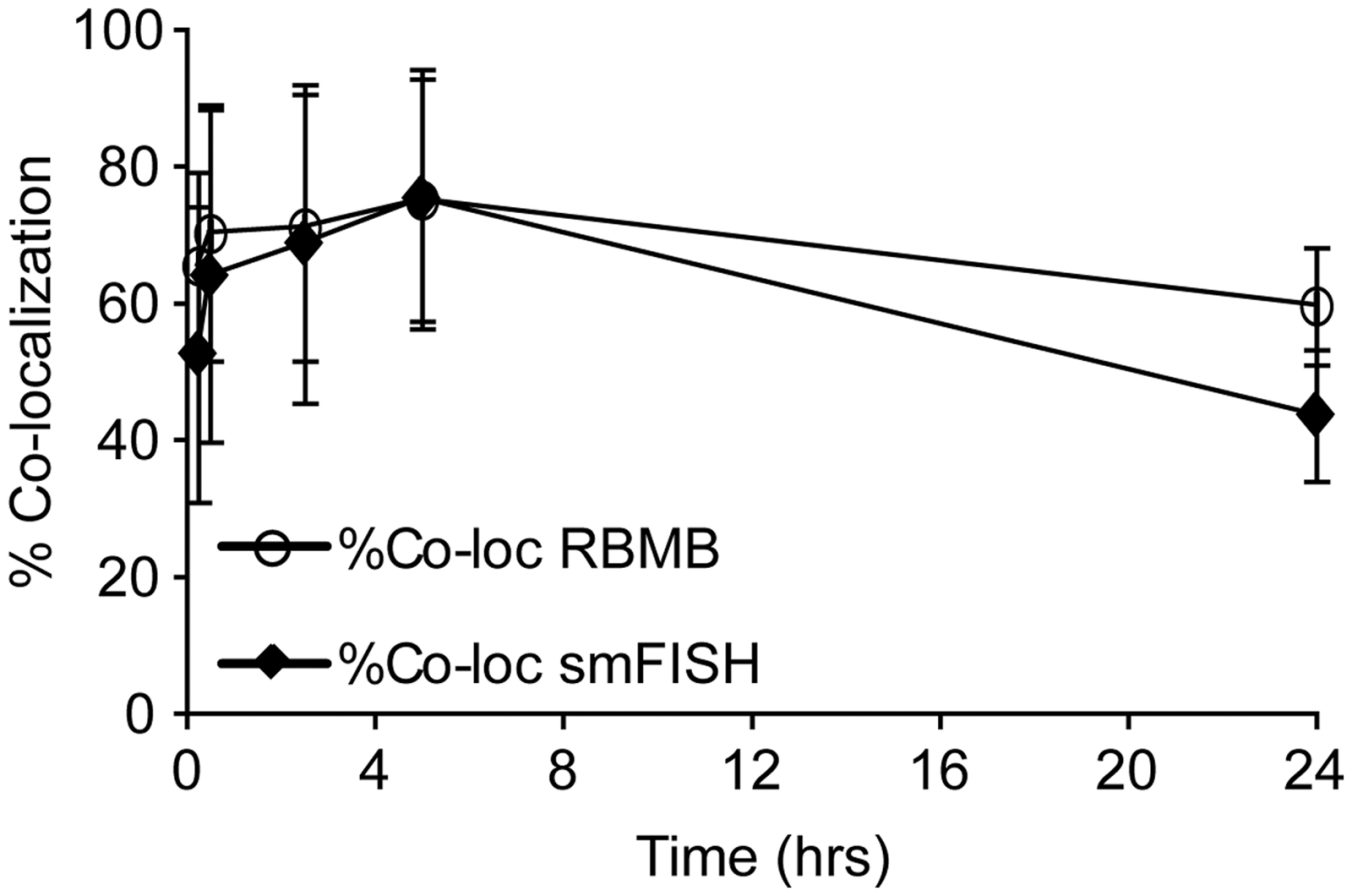
Ability to detect single RNA transcripts by RBMBs as a function of time after microporation. HT1080-GFP-96mer cells were microporated in the presence of 0.8 μM RBMBs and placed in the incubator for 15 min, 30 min, 2 h, 5 h or 24 h. The cells were then fixed and smFISH was performed. A custom Matlab program was used to determine the percent of RBMB-reporter signals that were colocalized with smFISH signals and the number of smFISH signals that were colocalized with RBMB-reporter signals for each time point, on a cell-by-cell basis. Each data point represents the mean measurement of colocalization (±standard deviation) for at least 20 cells, collected from at least two independent experiments.

To ascertain the time necessary for RBMBs to hybridize to target RNA and provide an accurate measure of the number of transcripts within individual cells, HT1080-GFP-96mer cells were fixed at various times following microporation in the presence of 0.8 µm RBMBs. Within 30 min, the colocalization was ∼65 and 70% for smFISH and RBMBs, respectively. This suggests that hybridization was fairly rapid, allowing for gene expression analysis to be conducted in a relatively short time frame. Measurements of gene expression seemed to be accurate for at least 5 h after microporation. However, by 24 h, only 60% of the RBMB signals colocalized with smFISH signals and only 44% of the smFISH signals colocalized with the RBMB signals (Figure 5). This decline in colocalization could be due to RBMB degradation at later time points, although this would contradict our previous findings, which have shown that RBMBs remain stabile for at least 24 h (31). Another possibility is that the RBMB probes become diluted during cell division. This would be consistent with our observations that only a fraction of cells exhibit poor colocalization, perhaps those that have undergone one or more cell divisions.

### Effect of reducing the number of engineered tandem repeats on RNA detection

**Figure 6.**
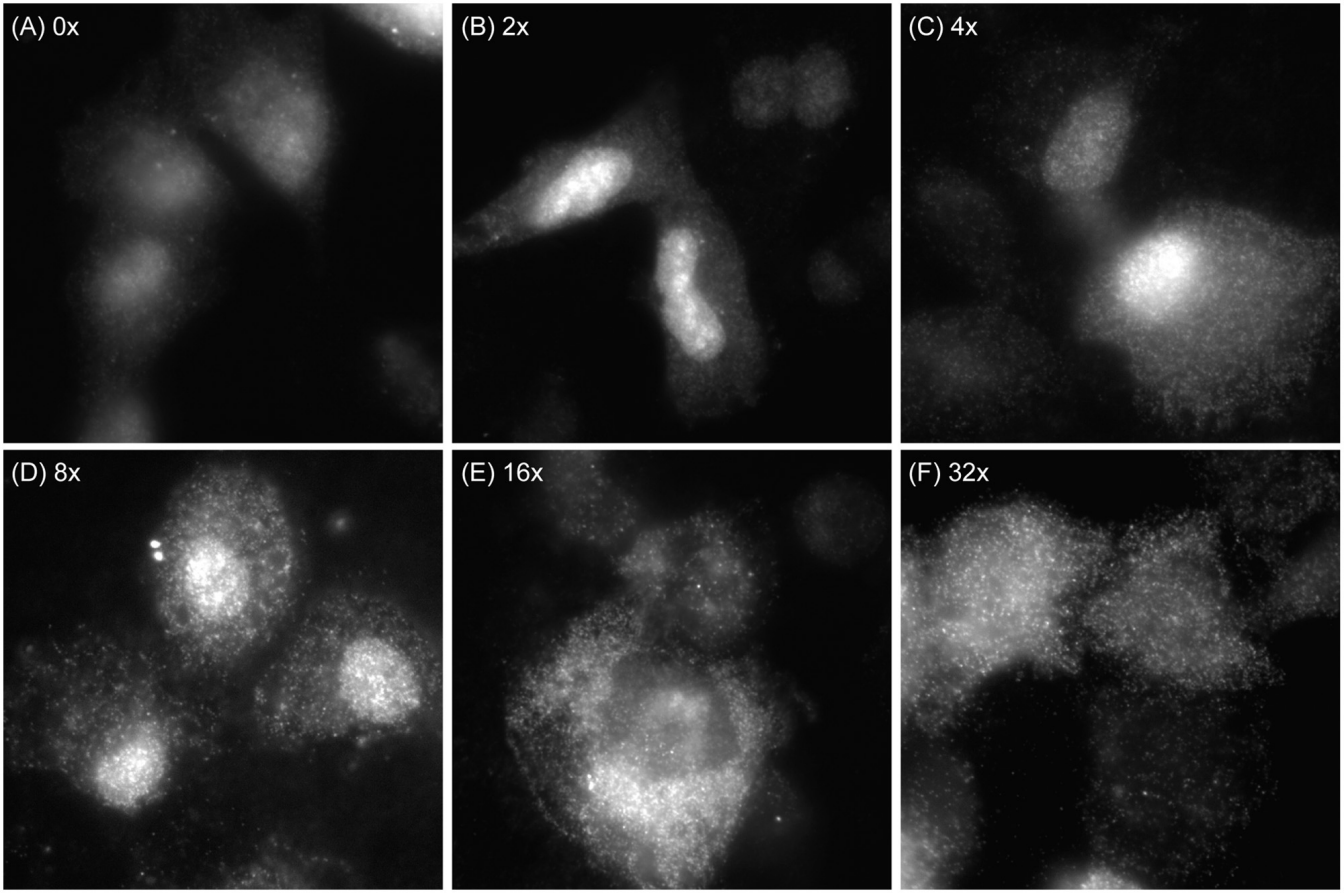
Detection of single RNA transcripts with varying number of RBMB binding sites. RNAs containing (**A**) 0, (**B**) 2, (**C**) 4, (**D**) 8, (**E**) 16 or (**F**) 32 RBMB-binding sites were produced by *in vitro* transcription. The RNA transcripts were then hybridized with RBMBs and microporated into HT1080 cells. After 1–3 h, the cells were fixed and fluorescent images of the RBMB reporter signals were acquired. Representative images are shown.

Because it can often be challenging to engineer a gene with 96-tandem repeats in the 3′-UTR and introduce it into cells, we investigated whether single RNA transcripts with fewer repeats could be detected in live and fixed cells. Specifically, RNAs containing between 2 and 32 RBMB binding sites were produced by *in vitro* transcription. The RNA transcripts were then hybridized with RBMBs and microporated into HT1080 cells. After 1–3 h, live cell streaming movies were acquired. Following live cell imaging, the cells were fixed and fluorescent images of the RBMB reporter signals were acquired. No processing steps were performed following fixation. It was found that individual RNA transcripts with as few as four RBMB binding sites could be observed moving within the nucleus and cytoplasm of living cells (Supplementary Movie S3), but as expected, the signal was not as pronounced as with 96-repeats. Photobleaching of the fluorescent signals was also more apparent, likely due to the smaller number of fluorescent dyes associated with each transcript. RNA transcripts with as few as four RBMB binding sites could also be detected in fixed cells (Figure 6). These results suggest that it may be feasible to detect endogenous RNAs with RBMBs, if four or more RBMBs are designed to hybridize the same RNA transcript.

### RBMBs versus traditional MBs

**Figure 7.**
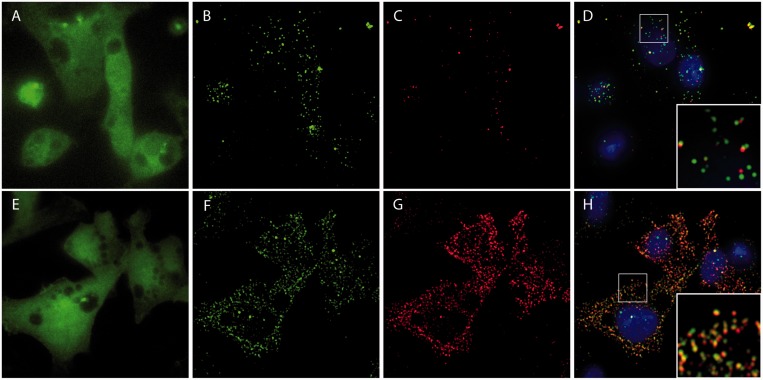
Comparison of conventional MBs and RBMBs in their ability to detect and quantify single RNA transcripts. HT1080-GFP-96mer cells were microporated in the presence of 0.8 μM MBs and placed in the incubator for 5 h. The cells were then fixed and smFISH was performed. Wide-field fluorescent images of (**A**) GFP, (**B**) smFISH probes (TMR) and (**C**) the MB-reporter dye (CF640R) were acquired. The images of the smFISH probes and the MB reporter dye shown are maximum intensity projections of 56-images within a z-stack. (**D**) A merged image that includes DAPI (blue) and the maximum intensity projections of smFISH probes (green) and the MB-reporter dye (red). Analogous studies were performed with 0.8 μM RBMBs. Wide-field fluorescent images of (**E**) GFP, (**F**) smFISH probes (TMR) and (**G**) RBMB-reporter dye (CF640R) were acquired. (D) A merged image that includes DAPI (blue) and the maximum intensity projections of smFISH probes (green) and the RBMB-reporter dye (red). A magnified view of the regions outlined by white boxes are shown in the inset of panels D and **H**.

Numerous studies have shown that traditional MBs can be used to measure gene expression in living cells (21–24); however, nuclear sequestration and nonspecific opening can interfere with the ability to sensitively detect target RNA (25–29). These shortcomings led to the development of RBMBs. Here, the ability of RBMBs and MBs to detect single RNA transcripts in HT1080-GFP-96mer cells was directly compared. Specifically, 0.8 µm of MBs or RBMBs were microporated into HT1080-GFP-96mer cells and smFISH was performed 5 h after microporation. Quantitative analysis of RBMB reporter and smFISH fluorescence revealed that both techniques provided similar estimates of RNA copy number, with ∼75% of the RBMB signals co-colocalized with smFISH signals and ∼75% of the smFISH signals colocalized with RBMB signals. In contrast, there were significantly fewer MB signals than smFISH signals. As a result, although 70% of the MB signals colocalized with smFISH signals, only 20% of smFISH signals colocalized with MB signals (Figure 7). These findings suggest that only a small fraction of the RNA transcripts are being detected by MBs. It is suspected that MB degradation, nuclear sequestration and/or nonspecific protein interactions reduced the actual concentration of MBs available for RNA hybridization. To confirm that differences in the ability of RBMBs and MBs to detect RNA transcripts in living cells did not stem from differences in probe functionality, thermal denaturation and hybridization kinetic assays were performed with both probes. It was found that RBMBs and MBs exhibited similar melting temperatures and hybridization on-rates (Supplementary Figure S4).

### Analysis of total cellular fluorescence in single cells

**Figure 8.**
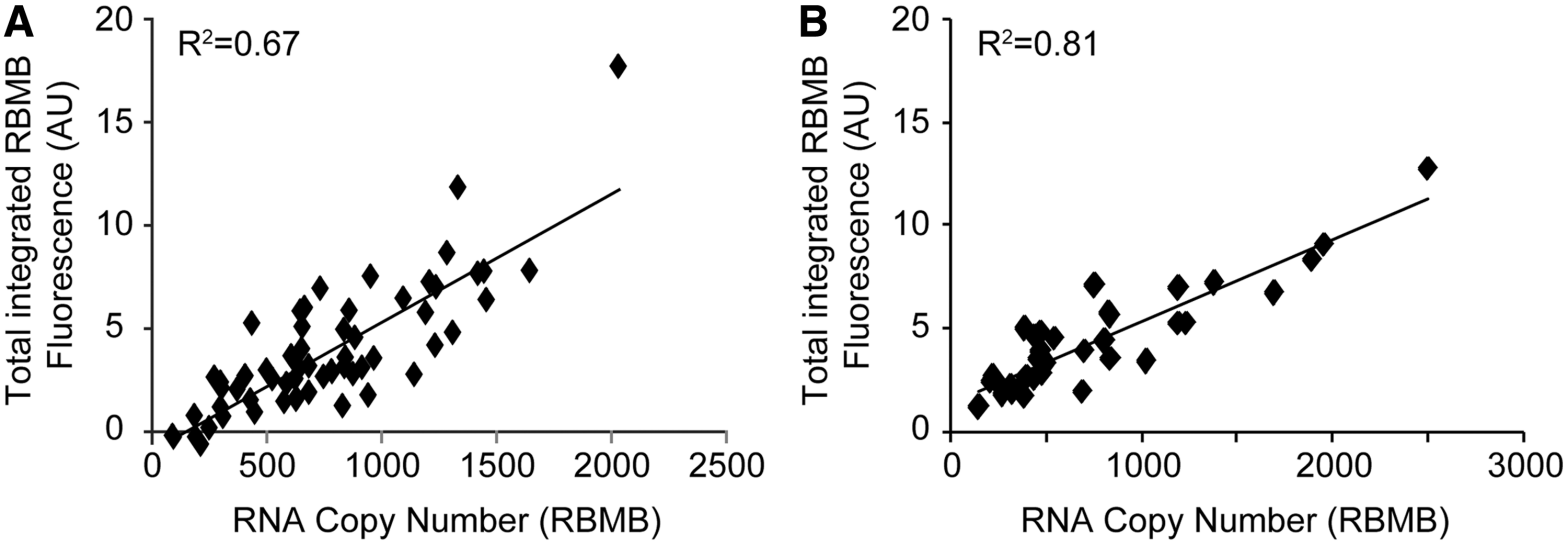
Comparison of total cellular fluorescence and RNA copy number. HT1080-GFP-96mer cells were microporated in the presence of 0.8 μM RBMBs and placed in the incubator for 5 h. The cells were then fixed and an image of a single focal plane was acquired using a 40× objection. A z-stack consisting of 56 images (0.2 μm increments) was also obtained using a 100× objective. (**A**) Measurements of total cellular fluorescence in the RBMB-reporter channel, obtained under lower magnification, was compared with RNA copy number per cell, which was quantified from the high magnification z-stacks. (**B**) Analogous studies were performed with CHO-GFP-96mer cells.

Although the ability to visualize and quantify individual RNA transcripts in single cells is valuable, single-molecule resolution is not always necessary. In many cases, single-cell analysis may actually be favored because it can be achieved under lower magnifications, requires the acquisition of only a single imaging plane and is more amenable for high-throughput analysis. To evaluate the ability of RBMBs to provide semiquantitative measurements of gene expression at the single-cell level, HT1080-GFP-96mer cells were fixed 5 h following the intracellular delivery of RBMBs via microporation. Images of single focal planes in the GFP, DAPI, TMR, RBMB-reporter and RBMB-reference channels were acquired using a 40× objective. Images were acquired in the TMR channel to provide an estimate for the level of autofluorescence in the RBMB-reporter channel, based on a previously determined relationship, as described in ‘Materials and Methods’ section. Measurements of the RBMB reference dye were used to correct RBMB-reporter fluorescence for cell-to-cell variations in RBMB delivery. The same cells were subsequently imaged using a 100× objective. Z-stacks consisting of 56 images were acquired in the RBMB-reporter channel and the total number of RNA transcripts was quantified in each cell. As expected, there was a linear correlation between the corrected RBMB-reporter fluorescence per cell and the absolute number of RNA transcripts (Figure 8A). Similar results were obtained in CHO-GFP-96mer cells (Figure 8B). It is speculated that the variability that is observed stems from imprecise measurements of autofluorescence and the low signal-to-noise in the RBMB-reference channel. Nonetheless, these studies provide initial evidence that using RBMBs for the high-throughput analysis of gene expression within single cells is a possibility.

## CONCLUSION

RBMBs can be used to image the movement of single RNA transcripts in living cells, when the target RNA is engineered to contain as few as four RBMB-binding sites. Moreover, RNA expression can be accurately quantified at both the single molecule level and at the single cell level. Measurements of gene expression can be acquired within 30 min, using a wide range of RBMB concentrations and in various cell types. These findings highlight the robustness and versatility of RBMBs as a tool for imaging RNA in live cells.

It should be noted that it has previously been shown that single engineered RNA transcripts can also be imaged in living cells through the coexpression of a GFP reporter that has been fused to the coat protein of bacterial phage MS2 (GFP-MS2) and an engineered RNA construct with tandem repeats of the MS2 binding site in the 3′-UTR.(36,37) However, while the GFP-MS2 system has facilitated the analysis of mRNA localization in a variety of organisms (38–41), the RBMB system described here has several important benefits. For example, the RBMB approach is highly robust, providing researchers with a great deal of flexibility in the experimental conditions. In particular, this method is relatively insensitive to the relative and total level of RNA expression and RBMB concentration. Other advantages include the ability to select organic fluorophores that are brighter and more photostable than fluorescent proteins. This makes it easier for labs with less microscopy expertise and poorer optics to image RNA transcripts. There are also more choices of far to near infrared dyes that can be selected to reduce background from autofluorescence. The quenching mechanism is also robust for RBMBs, which lowers background significantly compared with unquenched fluorescent proteins. The high signal-to-background allowed RNAs with as few as four bound RBMBs to be detected in living cells. The need for only four unique RBMBs to bind the same RNA target also potentially opens up an opportunity to detect endogenous RNA in living cells. Overall, we envision that the unique capabilities of RBMBs will open up many new avenues for RNA research.

## SUPPPLEMENTARY DATA

Supplementary Data are available at NAR Online: Supplementary Figures 1–4, and Supplementary Movies 1–3.

## FUNDING

Funding for open access charge: National Science Foundation CAREER Award (0953583) and the National Institute of Health (NCI) [R21 CA116102 and R21 CA125088].

*Conflict of interest statement*. M.B. and L.H. are employed by IDT, which offers oligonucleotides for sale similar to some of the compounds described in the manuscript. IDT is, however, not a publicly traded company and he personally does not own any shares/equity in IDT.

## Supplementary Material

Supplementary Data
