# Supplementary material for: Quantitative assessment of ratiometric bimolecular beacons as a tool for imaging single engineered RNA transcripts and measuring gene expression in living cells
Source: Nucleic Acids Res. 2013 Jun 27;41(15):e152. doi: 10.1093/nar/gkt561 (PMC3753654; doi:10.1093/nar/gkt561)
Supplement: Supplementary Data [file supp_41_15_e152__index.html]

Quantitative assessment of ratiometric bimolecular beacons as a tool for imaging single engineered RNA transcripts and measuring gene expression in living cells — Supplementary Data 

# Quantitative assessment of ratiometric bimolecular beacons as a tool for imaging single engineered RNA transcripts and measuring gene expression in living cells

## 

files

**Files in this Data Supplement:**

- Supplementary Data - pdf file
- Supplementary Data - avi file
- Supplementary Data - avi file
- Supplementary Data - avi file
