# Supplementary material for: Quantitative assessment of ratiometric bimolecular beacons as a tool for imaging single engineered RNA transcripts and measuring gene expression in living cells
Source: Nucleic Acids Res. 2013 Jun 27;41(15):e152. doi: 10.1093/nar/gkt561 (PMC3753654; doi:10.1093/nar/gkt561)
Supplement: Supplementary Data [file supp_gkt561_nar-01280-met-g-2013-File013.pdf]

SUPPLEMENTARY INFORMATION for

Quantitative Assessment of Ratiometric BiMolecular Beacons as a Tool for Imaging Single Engineered RNA Transcripts and Measuring Gene Expression in Living Cells

Xuemei Zhang[1], Yang Song[1], Akash Shah[1], Virzhiniya Lekova[2], Arjun Raj[1], Ling Huang[3], Mark A. Behlke[3] and Andrew Tsourkas[1,*]

[1]Department of Bioengineering, University of Pennsylvania, Philadelphia, PA 19104

[1]Department of Biology, University of Pennsylvania, Philadelphia, PA 19104

[3]Integrated DNA Technologies, Inc., Coralville, IA 52241

SUPPLEMENTARY FIGURES:

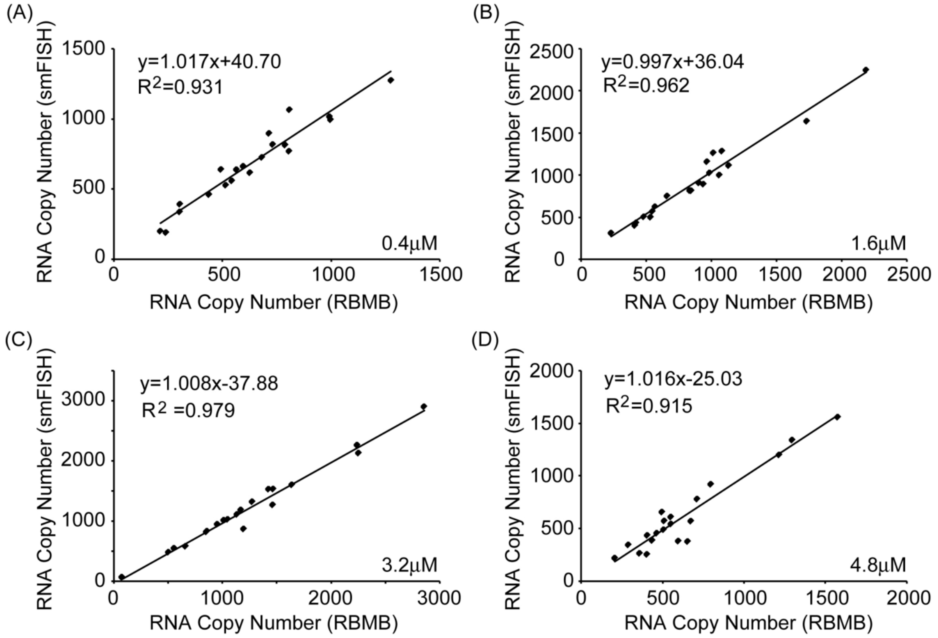

Figure S1. Comparison of RNA copy number as determined by smFISH and with RBMBs. HT1080-GFP-96mer cells were microporated in the presence of RBMBs and placed in the incubator for 5hr. The concentration of RBMBs used during microporation was (A) 0.4μM (B) 1.6μM (C) 3.2μM or (D) 4.8μM. The cells were then fixed and smFISH was performed. (A) A custom Matlab program was used to determine the percent of RBMB-reporter signals that were co-localized with smFISH signals and the number of smFISH signals that were co-localized with RBMB-reporter signals. (B) Measurements of RNA copy number, as determined by smFISH and RBMBs, were also compared.

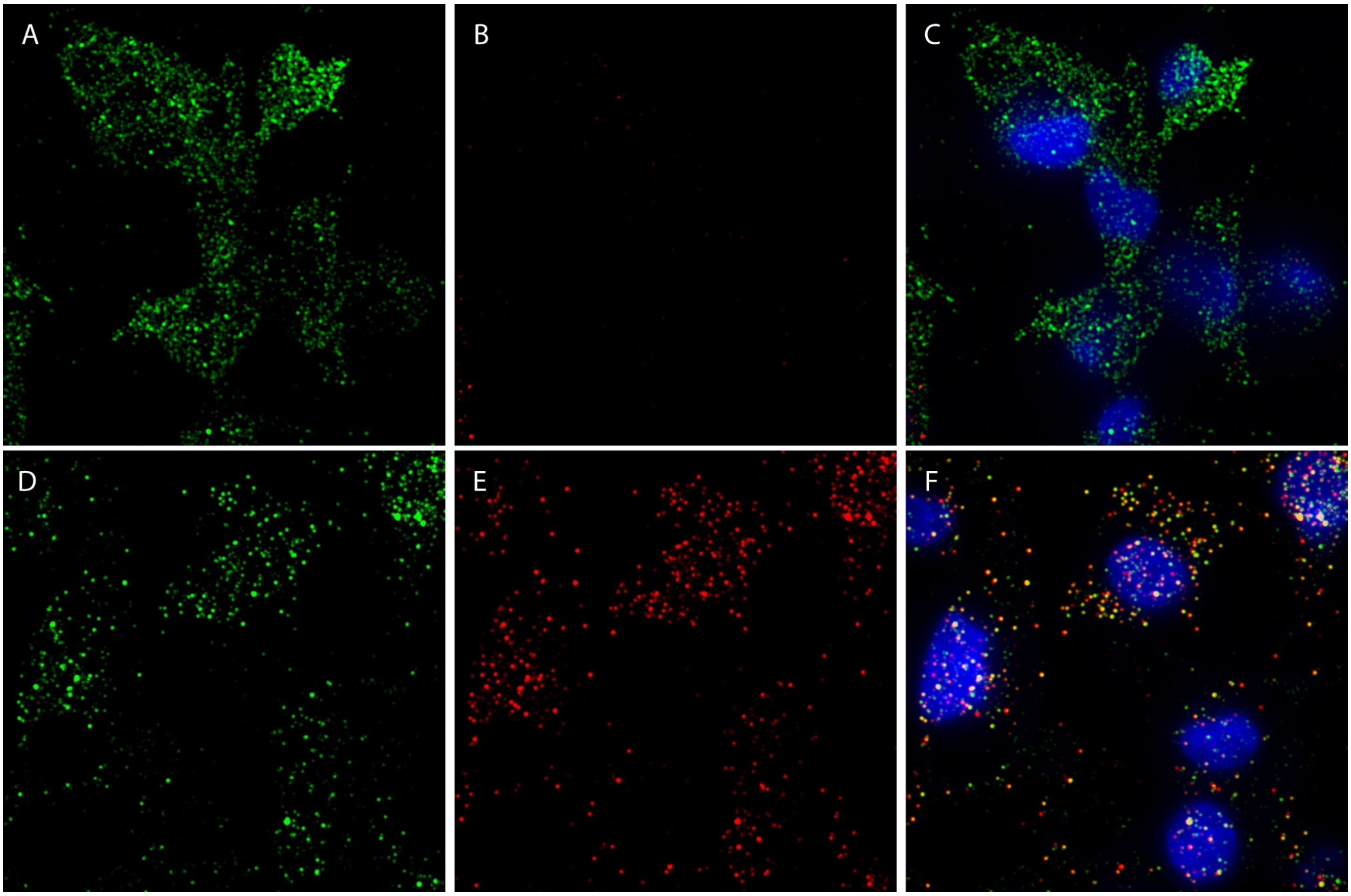

Figure S2. Positive and negative controls for smFISH and analysis of RNA copy number. Negative control studies were performed by microporating HT1080-GFP cells (i.e. no repeats) in the presence of 0.8μM RBMBs and placing the cells in the incubator for 5hr. The cells were then fixed and smFISH against the GFP coding sequence was performed. Wide-field fluorescent images of (A) smFISH probes (TMR), and (B) RBMB-reporter dye (CF640R) were acquired. The images shown are maximum intensity projections of 56-images within a z-stack. (C) A merged image that includes DAPI (blue) and the maximum intensity projections of smFISH probes (green) and the RBMB-reporter dye (red). Positive control studies were performed by microporating HT1080-GFP-96mer cells in the absence of RBMBs and placing the cells in the incubator for 5hrs. The cells were then fixed and smFISH against the GFP coding sequence and the 96-repeats was performed. Wide-field fluorescent images of (A) smFISH probes against GFP (TMR), and (B) smFISH probes against the 96-repeats (Alexa594) were acquired. The images shown are maximum intensity projections of 56-images within a z-stack. (C) A merged image that includes DAPI (blue) and the maximum intensity projections of both smFISH probes (green).

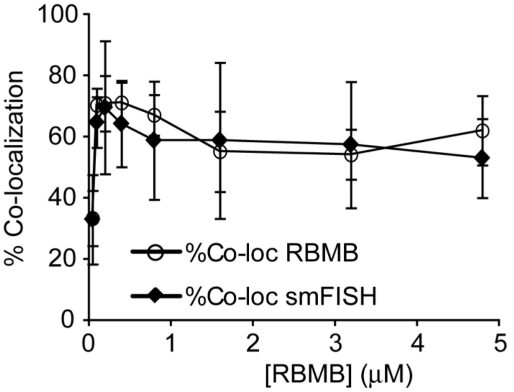

Figure S3. Assessing the accuracy of RBMB-based measurements of RNA copy number. CHO-GFP-96mer cells were microporated in the presence of RBMBs and placed in the incubator for 5hr. The concentration of RBMBs was varied from 0.05μM to 4.8μM. The cells were then fixed and smFISH was performed. (A) A custom Matlab program was used to determine the percent of RBMB-reporter signals that were co-localized with smFISH signals and the number of smFISH signals that were co-localized with RBMB-reporter signals, on a cell-by-cell basis. Each data point represents the mean measurement of co-localization (± standard deviation) for at least 20 cells, collected from at least two independent experiments.

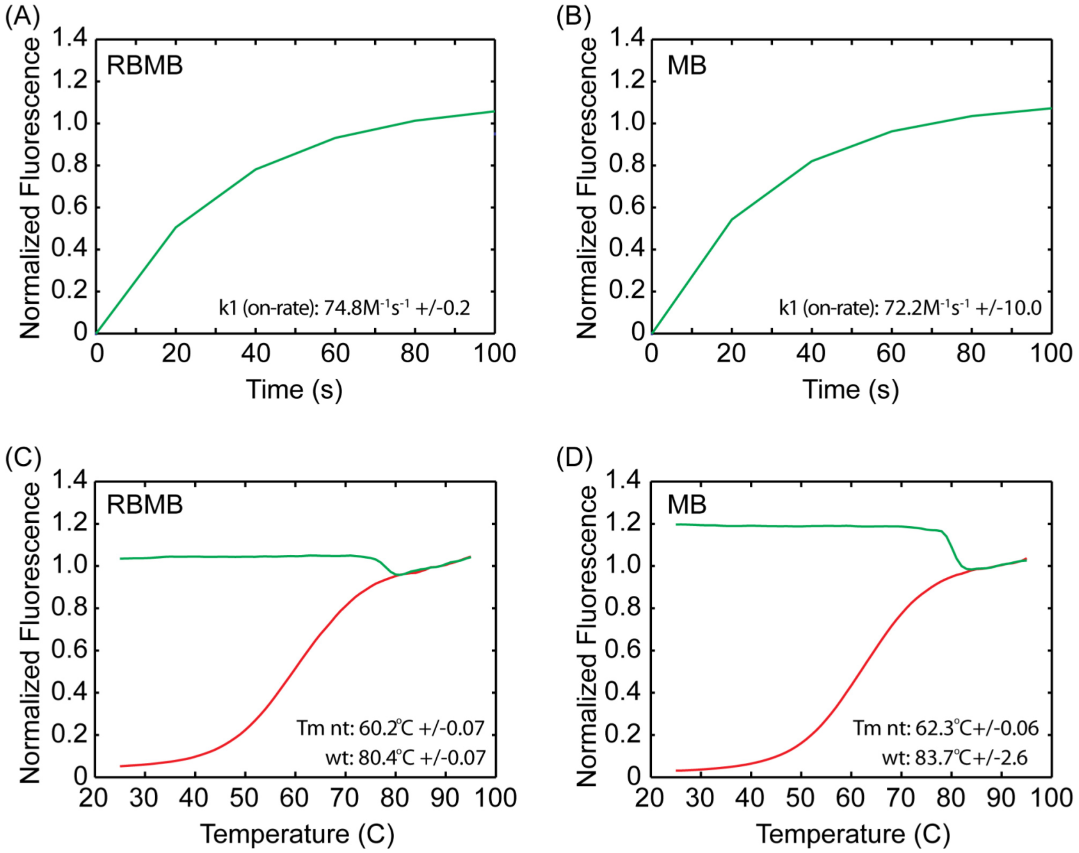

Figure S4. Kinetic and melting temperature analysis of RBMBs and MBs. Hybridization kinetics between (A) RBMBs and complementary RNA targets and (B) MBs and complementary targets were determined by mixing 50nM probe with 500nM target and recording fluorescence as a function of time. Heat denaturation profiles of (C) RBMB-RNA hybrids and (D) MB-RNA hybrids were acquired by first mixing 50nM probe with 500nM complementary RNA target (green line). These samples were then heated to 95°C and the temperature was reduced to 25°C at 1°C increments. The temperature was held at each temperature increment for three minutes and fluorescence was recorded. Analogous studies were performed in the absence of RNA target (red line). The heat denaturation profiles were used to determine the melting temperature of RBMBs and MBs in the presence (wT) and absence of target (nT).
